# Medical Plants and Immunological Regulation

**DOI:** 10.1155/2018/9172096

**Published:** 2018-06-12

**Authors:** Cheng Xiao, Qingdong Guan, Yong Tan, Lifei Hou, Wuxiang Xie

**Affiliations:** ^1^Institute of Clinical Medicine, China-Japan Friendship Hospital, Beijing 100029, China; ^2^Cellular Therapy Laboratory, CancerCare Manitoba, Winnipeg, MB, Canada R3A 1R9; ^3^Institute of Basic Research in Clinical Medicine, China Academy of Chinese Medical Sciences, Beijing 100700, China; ^4^Program in Cellular and Molecular Medicine, Department of Pediatrics, Boston Children's Hospital, Harvard Medical School, Boston, MA 02115, USA; ^5^Peking University Clinical Research Institute, Peking University Health Science Center, Beijing 100191, China

Immune regulation is crucial for the maintenance of immune system homeostasis. The abnormalities of immune regulation are involved in many diseases and disorders. Increasing studies show that medical plants, corresponding monomers, and formulas exert immunomodulatory effects, such as stimulatory effects on immune cells, immune organs, and cytokine production, as well as inhibitory effects on inflammation, allergy, and autoimmune disease. In this special issue, we present original research articles as well as review papers on medical plants and immunological regulation.

Plant monomers are major effective constituents of medical plants. Studies on monomers are greatly increased in recent years because they have specific molecular structure and are easier to be used in mechanism and molecule target research [1, 2]. The imbalance of T cell subsets plays an important role in rheumatoid arthritis (RA). IFN-*γ* derived from Th1 cell predominates during the induction and acute phases of RA. S. Xu et al. investigated the antiarthritic potential of ethyl caffeate (ECF) isolated from *Elephantopus scaber* L. in collagen-induced arthritis (CIA) and found that ECF ameliorated CIA by suppressing Th1 response and IFN-*γ* signaling pathway. Acute lung injury (ALI) is closely associated with the increased production of inflammatory mediators including cytokines (TNF-*α*, IL-1*β*, and IL-6), especially produced by alveolar macrophages and neutrophils. A. R. Pinheiro et al. assessed the anti-inflammatory potential of the ethyl acetate fraction (EAFPg) extracted from pomegranate leaf in a mouse model of LPS-induced acute lung injury and proved that EAFPg could prevent inflammatory disorders in ALI. Matrine is a bioactive component extracted from *Sophora flavescens*. H. Fan et al. explored the therapeutic effect of matrine on CRC and found that matrine treated CRC via inhibition of HMGB1 signaling characterized by the downregulation of IL-6, TNF-*α*, and HMGB1.

The study on bioactive fraction is a hotspot of medical plant research [3]. Flavonoids from medical plants are considered as powerful immunomodulatory agents [4]. D. Kelepouri et al. reviewed the role of flavonoids in inhibiting Th17 responses in RA and experimental autoimmune arthritis. D. Shi et al. explored the immunomodulatory effect of flavonoids from blueberry leaves (FBL) in lipopolysaccharide- (LPS-) stimulated RAW 264.7 cells and found that the immune regulatory effects of FBL was through suppressing TNF via the NF-*κ*B signal pathway. Besides, immunomodulatory activities of single medical plant were explored in this special issue. *Ophiocordyceps lanpingensis* (OL), a popular tonifying Chinese medical plant, has been used to treat renal inadequacy. Y. Zhang et al. investigated the protective effects of OL in acute renal failure (ARF) and found that OL could ameliorate renal dysfunction in glycerol-induced ARF in mice by inhibiting oxidative stress and enhancing IgG immune response.

Multiherbal formulas based on traditional Chinese medicine have been scientifically verified for use in complementary and alternative therapy for the diseases resulted from abnormal immunomodulation, and they act on multiple targets and exert synergistic therapeutic efficacies [5, 6]. The clinical application of these formulas has attracted more and more scientific attentions. This special issue presented some studies which explored pharmacological mechanism of the formulas. Mahuang Fuzi Xixin decoction (MFXD), a famous Chinese formula, has been widely used in treating allergic rhinitis (AR). M. Ren et al. confirmed its therapeutic effect on allergic inflammation by regulating Th1 and Th2 immune responses in a rat model of OVA-induced AR. J. Zhou et al. investigated the effects of modified Danggui Buxue Tang (DGBX, a Chinese formula) on the regulation of the balance between proliferation and apoptosis of hematopoietic stem cells (HSCs) due to the aberrant immune response in a mouse model of aplastic anemia. The results indicated that DGBX could attenuate IFN-*γ* production through interfering in SLAM/SAP signaling and distribution of T-bet in T cells, exert immunosuppressive effects by modulating the activation of the Stat/JAK/IRF-1 pathway, restrain cell apoptosis by intervening Fas-dependent pathway, and eventually attenuate immune-mediated destruction of HSCs. Pien Tze Huang (PZH), a classical Chinese patent formula, has been proved to have anti-inflammatory, neuroprotective, and immune regulatory effects. X. Qiu et al. investigated its therapeutic effects on experimental autoimmune encephalomyelitis (EAE) rats and found that PZH not only ameliorated the clinical severity of EAE rats but also remarkably reduced inflammatory cell infiltration in the central nervous system (CNS) of EAE rats, as well as significantly decreased the levels of IL-17A, IL-23, CCL3, and CCL5 in serum and the levels of p-P65 and p-STAT3 in the CNS. Gen Tong Ping (GTP) granule, another Chinese patent formula, has been widely used in curing cervical spondylotic radiculopathy (CSR). W. Sun et al. predicted the mechanism of GTP treating CSR using network pharmacology approach firstly and found that PPAR-*γ* pathway might be involved in it. Then, the rat model of CSR induced by spinal cord injury (SCI) was used to verify the prediction. The results demonstrated that GTP modulated the PPAR-*γ* pathway by inhibiting the immune inflammatory response and apoptosis as well as by protecting the cytoskeletal integrity of the spinal cord, ultimately played a neuroprotective role in CSR.

This special issue also presents constructive reviews about using medical plants to treat common and frequently occurring immune inflammatory diseases. Atherosclerosis is a chronic inflammatory disease caused by dyslipidemia and mediated by both innate and adaptive immune responses. Y. Ren et al. systematically reviewed medical plants that act as immunomodulatory agents of suppressive function on cytokine production in atherogenesis. The immune factors play important regulatory roles in the occurrence of osteoporosis. H. Zhao et al. provided a general overview on the immune regulation mechanisms of medical plants and corresponding monomers in the prevention and treatment of osteoporosis. Medical plants have been widely used to treat diabetes. Z. Gao et al. reviewed the pharmacological mechanisms of representative medical plants, monomers, and formulas treating diabetes and proposed that these remedies improve glucose homeostasis through the “Bacteria-Mucosal Immunity-Inflammation-Diabetes” Axis. This axis was considered a “line” to string most antidiabetic agents together and might provide new perspectives and strategies for future research on diabetes and the development of hypoglycemic drugs.

Taken together, the articles in this special issue provide insights into medical plants and immunological regulation. In the future, an extensive investigation is required with respect to their precise mechanisms at the systemic, cellular, and molecular levels and extension to a large-scale clinical trial. Given the identified immunomodulatory effects of medical plants, it is interesting to design future therapeutic strategies for immune inflammatory diseases with a synergistic combination of medical plants and conventional therapies. We hope that researchers enjoy the articles of this special issue.
